# Deformation Monitoring of Metro Tunnel with a New Ultrasonic-Based System

**DOI:** 10.3390/s17081758

**Published:** 2017-08-01

**Authors:** Dong-Sheng Xu, Yu-Meng Zhao, Hua-Bei Liu, Hong-Hu Zhu

**Affiliations:** 1 School of Civil Engineering and Mechanics, Huazhong University of Science and Technology, 1037 Luoyu Rd., Wuhan 430074, China; ymzhao1114@gmail.com (Y.-M.Z.); hbliu@hust.edu.cn (H.-B.L.); 2 School of Earth Sciences and Engineering, Nanjing University, Nanjing 210023, China

**Keywords:** tunnel deformation measurement, ultrasonic, wireless, redundant ultrasonic information

## Abstract

With the rapid construction of metro tunnels in many metropolises, a fast and convenient solution to capture tunnel deformation is desired by civil engineers. This contribution reports an automatic and wireless tunnel deformation monitoring system using ultrasonic transducers. A processing algorithm of the redundant ultrasonic information (RUI) approach is proposed to improve measurement accuracy. The feasibility of this tunnel deformation monitoring method is carefully examined with various probe angles, distances, and surrounding temperature variations. The results indicate that high accuracy can be achieved with different coefficients for various probe angles and sensor distances, as well as temperatures. In addition, a physical tunnel model was fabricated to verify the new processing algorithm of the RUI approach for a wireless tunnel deformation sensing system. The test results reveal that average measurement errors decreased from 7% to 3.75% using the RUI approach. Therefore, it can be concluded that the proposed approach is well suited to the automatic detection of critical conditions such as large deformation events in metro tunnels.

## 1. Introduction

With the accelerated urbanization and population growth, numerous tunnels have been constructed to fit the needs of the increasing demands of transportation, power transmission, water supply, and drainage. In China alone, 23 cities have metros up to 2735 km, with a further 2853 km under construction [1]. During operation, lateral and transversal deformations of tunnel structures may occur when there are adjacent geotechnical construction activities, such as deep excavation, pile installation, tunneling, and dewatering [2]. The excessive tunnel deformation will diminish the effective usage of metro systems or even cause unexpected disasters. Such deformation leads to additional joint stresses and redistribution of internal forces in structural members, accompanied by long-term uneven settlement of the ground surface. All of these phenomena are harmful to the normal operation of metro tunnels and adjacent infrastructures [3,4,5,6,7].

The great numbers of tunnels under construction or in service demand the automatic monitoring of tunnel deformations. Many methods have been developed, among which the most traditional one is to utilize the surveyor’s level and total stations [8,9]. The laser and optical fibers have also been used recently [2,10,11]. However, all of these technologies have certain drawbacks. For instance, the leveling surveyor method require workers to operate, and hence it will be hard to reach some regions such as the tunnel roofs (let alone the difficulty of monitoring the deformation automatically and continuously). Colored, shiny, or transparent surfaces, or adverse ambient conditions such as dust, dirt, or fog are particularly challenging for optical sensors [12]. Besides, optical fibers installed in the tunnels are fragile and the overall cost is higher than that of conventional technologies.

This study investigates the performance of deformation monitoring of tunnel cross sections using ultrasonic transducers, which is an innovative method for tunnels. Ultrasonic can realize wireless measurement of distance and the ultrasonic transducers are cheap for engineering applications [13]. These transducers have the potential to perform continuous and automatic measurement of tunnel deformations. They will not interfere with transportation when applied to an in-service traffic tunnel. However, due to the adverse environments encountered during tunnel monitoring, the measurement accuracy of an ultrasonic sensing system is affected by the orientation of the transmitter-receiver ultrasonic due to tunnel deformations, and may also be influenced by temperature changes. These factors are carefully examined in this study. In addition, a processing algorithm, i.e., the redundant ultrasonic information (RUI) approach, is proposed for the ultrasonic sensing system. The ultrasonic sensor arrangement for a tunnel cross section is also described in this study. Finally, the proposed RUI approach, together with the whole monitoring system, is validated in a physical tunnel model test.

## 2. Methodology

### 2.1. Principles of Sensor Coordinates Determination

**Figure 1:**
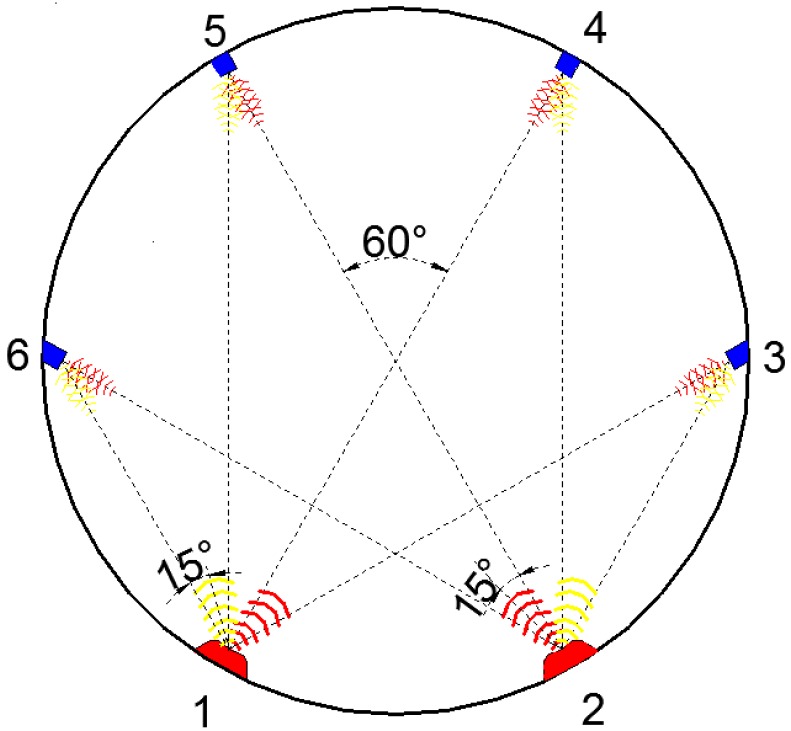
Configuration of the ultrasonic transducer system for deformation monitoring.

**Figure 2:**
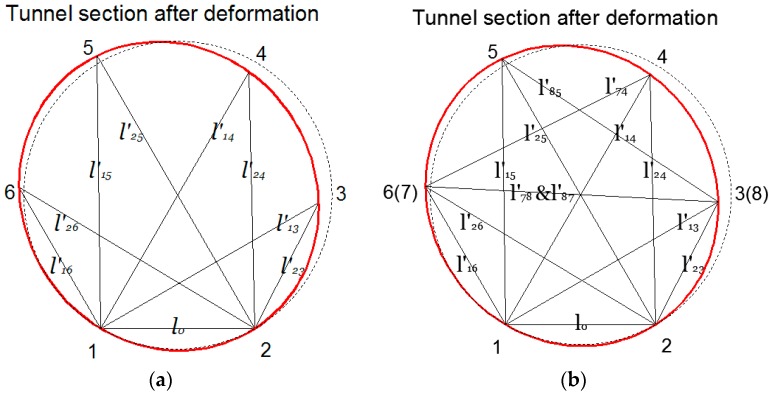
Algorithm of tunnel deformation monitoring: (**a**) deformation of the tunnel cross section without the RUI algorithm; (**b**) deformation of the tunnel cross section by using the RUI algorithm.

Here we developed a kind of ultrasonic transducer and the corresponding computing method to monitor the deformation of the tunnel cross section. As shown in Figure 1, two ultrasonic transmitters and four ultrasonic receivers are fixed at the circular cross section of a tunnel. Transducers 1 and 2 are the transmitters, and Transducers 3, 4, 5, and 6 are the receiving ultrasonic transducers. Both Transducers 1 and 2 are two-oriented ultrasonic transmitters that emit ultrasonics from each to all the receivers. All the transducers are evenly distributed on the tunnel cross section with 60° central angles between each other. The orientation of the receivers direct at the transmitters, respectively, while the transmitters have two probes with an angle of 60°. The demonstrated configuration shows the minimum transducers needed to determine the position of each transducer. It was assumed that the global coordinates of transducers 1 and 2 will not change. The distance before deformation between transmitter *i* and receiver *j* is labeled as *l_ij_* , and after deformation is expressed as *l’_ij_*. The detailed information is illustrated in Figure 2. All the *l_ij_* and *l’_ij_* shall be measured by ultrasonic transducers. Compared to the configuration with the truss theory in structural mechanics, if Transducers 1 and 2 are fixed on the ground, the coordinate of each node can be determined. When the distances before and after tunnel deformation have been measured, the change of coordinate for each transducer can be determined, and so change of the coordinate of the tunnel at the points that the transducers are located. It should be noticed that in this configuration, the rigid body displacement of the tunnel cross section (*x*, *y,* and *θ*) was not considered, which means that this system only measures the relative displacements of Transducers 1 to 6.

### 2.2. The RUI Method

A method to improve the measurement accuracy of tunnel deformation is discussed here. The widely used least square method will yield relatively larger errors; thus, a new method by using redundant ultrasonic information (RUI) was proposed, which can overcome the limitation of the traditional method, as discussed above. The concept of the RUI method was put forward by Jiang et al. [14]. The distance measured by ultrasonic wave propagation can be written as:

(1) $$L_{n}=\nu \cdot t_{p}$$

where *L_n_* represents the measurement of the distance from the *n*th sensor, and *ν* represents the ultrasonic velocity in the measurement. *ν* can be witnessed as a constant if the required accuracy is not very high. *t_p_* is the TOF (time of flight) of the *n*th receiver. The measured *t_p_* has some errors due to the lagging errors of ultrasonic wave and electric circuits, and thus *t_p_* can be written as:

(2) $$t_{p}=t_{c}t_{cn}^{\prime }-t_{e}-t_{d}$$

where *t_c_* is the unit time of the measurement count, *t’_cn_* is the counts of the *n*th receiver, *t_e_* is the wave lagging error which is caused by the insensitivity of the detection of the first coming receiving waves, and *t_d_* is the electric circuit lagging error. The electric circuit lagging error is negligible compared with the wave lagging error [14]. Thus, the error of the measured distances is:

(3) $$e=t_{e}\cdot \nu$$

where *e* is the distance lagging error. The frequency of the ultrasonic system is 40 kHz, and thus the wavelength is approximately 8.5 mm by taking ultrasonic velocity *v* = 340 m/s. In this study, the whole wave lagging error *e* should between 0 and 42.5 mm (5*λ*) [14]. When the wave lagging error of the ultrasonic receivers is consistent, the wave lagging error *e* can be determined and eliminated.

**Figure 3:**
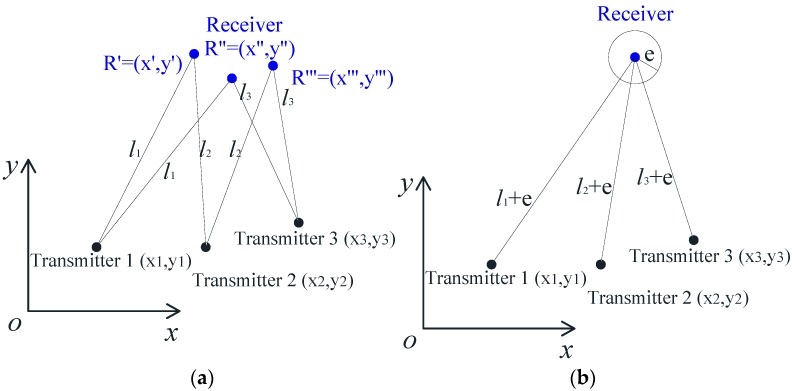
Illustrations of the RUI method: (**a**) dispersion of single redundancy information; (**b**) fusion of single redundancy information.

In Figure 3, it is supposed that three transmitters are located at coordinates (*x*_1_, *y*_1_), (*x*_2_, *y*_2_), and (*x*_3_, *y*_3_). *L*_1_, *L*_2_, *L*_3_ are the measured distance with ultrasonic wave propagations. The coordinates of point *R*(*x*, *y*) can be determined based on every two results of the three ultrasonic sensors. Thus, three different coordinates of *R*(*x*, *y*) will be computed, as shown in Figure 3a. According to the above discussions, the dispersion of computing *R*(*x*, *y*) is mainly caused by the ultrasonic transmitting distance error *e*. Although the major measurement error is caused by wave lagging, the errors induced by the synchronization of generation and acquisition may also result in dispersion. In this study, a fast micro-chip (STC12) with a time-clock 22.1 MHz and a cycle-per-instruction of 0.045 μs was used, which could largely decrease measurement errors induced by synchronization. In addition, this study adopted an automatic gain control (AGC) amplifier to minimize measurement errors.

For different measuring distances, the wave lagging errors will be consistent. Thus, the coordinate of *R*(*x*, *y*) could be computed from Equation (4):

(4) $$\{\begin{array}{c} {\left(x-x_{1}\right)}^{2}+{\left(y-y_{1}\right)}^{2}={\left(L_{1}+e\right)}^{2}\\ {\left(x-x_{2}\right)}^{2}+{\left(y-y_{2}\right)}^{2}={\left(L_{2}+e\right)}^{2}\\ {\left(x-x_{3}\right)}^{2}+{\left(y-y_{3}\right)}^{2}={\left(L_{3}+e\right)}^{2}\\ x^{2}+y^{2}=L^{2} \end{array}$$

**Figure 4:**
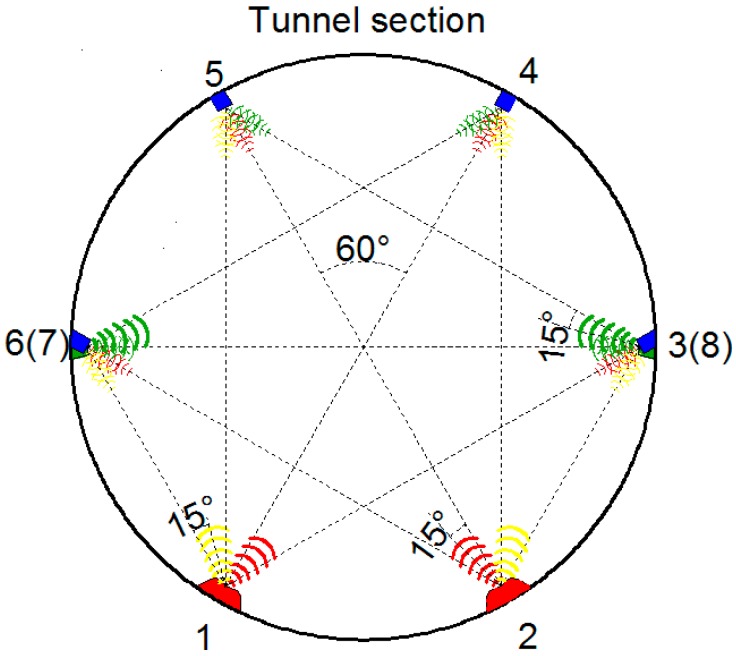
Configuration of the ultrasonic transducer system for tunnel deformation monitoring utilizing special fusion methods for redundancy ultrasonic information (RUI).

To apply the RUI method to the tunnel deformation monitoring system, two extra ultrasonic transmitters were used and mounted at point 7 and 8, as shown in Figure 4; thus, four more distances (*l*_74_, *l*_85_, *l*_78_ and *l*_87_) can be measured. Since the receiver was the same, the coordinates of sensor 6, 3, 4, and 5 in Figure 2 can be expressed as follows:

(5) $$\{\begin{array}{c} {\left(x_{6}-x_{1}\right)}^{2}+{\left(y_{6}-y_{1}\right)}^{2}={\left(L_{16}+e_{1}\right)}^{2}\\ {\left(x_{6}-x_{2}\right)}^{2}+{\left(y_{6}-y_{2}\right)}^{2}={\left(L_{26}+e_{1}\right)}^{2}\\ {\left(x_{6}-x_{8}\right)}^{2}+{\left(y_{6}-y_{8}\right)}^{2}={\left(L_{86}+e_{1}\right)}^{2} \end{array}$$

Correspondingly, the receiver at (*x*_3_, *y*_3_) would also receive three beams of ultrasonic from transmitter 1, 2, and 7. Thus, other three sets of equations could be acquired:

(6) $$\{\begin{array}{c} {\left(x_{3}-x_{1}\right)}^{2}+{\left(y_{3}-y_{1}\right)}^{2}={\left(L_{13}+e_{2}\right)}^{2}\\ {\left(x_{3}-x_{2}\right)}^{2}+{\left(y_{3}-y_{2}\right)}^{2}={\left(L_{23}+e_{2}\right)}^{2}\\ {\left(x_{3}-x_{7}\right)}^{2}+{\left(y_{3}-y_{7}\right)}^{2}={\left(L_{73}+e_{2}\right)}^{2} \end{array}$$

Given that (*x*_1_, *y*_1_) and (*x*_2_, *y*_2_) were known and were kept unchanged during the test as well as (*x*_6_, *y*_6_) = (*x*_7_, *y*_7_) and (*x*_3_, *y*_3_) = (*x*_8_, *y*_8_), the Equations (6) and (7) can be used to solve the coordinates (*x*_6_, *y*_6_), (*x*_3_, *y*_3_) and the wave lagging errors *e*_1_ and *e*_2_. When the coordinates of sensor 3 (8) and 6 (7) have been determined, the location of sensor 5 and 6 could be easily obtained utilizing the similar equations of Equation (6).

## 3. Test Results and Analysis

### 3.1. Ultrasonic Distance Measurement System

**Figure 5:**
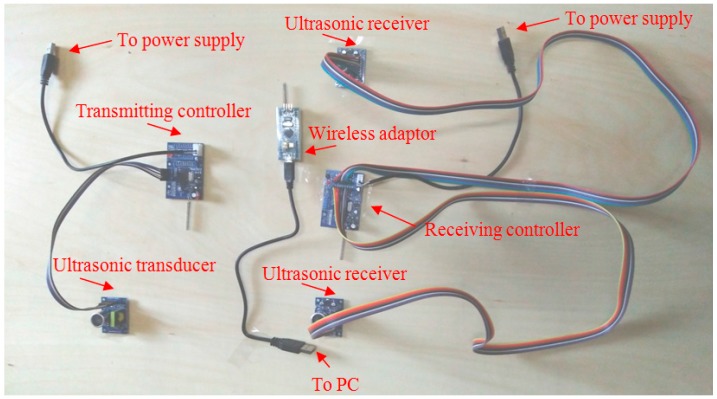
Photograph of the ultrasonic transducer system for tunnel deformation monitoring.

Figure 5 shows the main components of the ultrasonic-based monitoring system. The working principle is briefly described as follows: the command to emit ultrasonic was first generated at the PC terminator and sent to the wireless adaptor via a USB port. The communication between the adaptor and the transmitting controller was wireless. The transmitting controller was an enhanced micro-computer that ordered the transmitter to send ultrasound at 40 kHz via data wires. The ultrasonic receiver would then receive the signals and sent data via wires to the receiving controller. The communication between the receiving controller and the adaptor was wireless. Finally, the feedback was recorded in a personal computer and manifested as measured distance.

A prototype of a tunnel was constructed to illustrate the application of the ultrasonic distance measurement system. The tunnel model with 1.7 m diameter was made in wood so that the large tunnel deformation could be achieved and controlled easily. All the transducers were mounted on the tunnel model, as described in Figure 1, and points 1 and 2 were firmly fixed so that there was no displacement of these two points. The tunnel was then deformed. The distances between sensors were measured before and after deformation and the changes of distance were calibrated by LVDTs.

**Figure 6:**
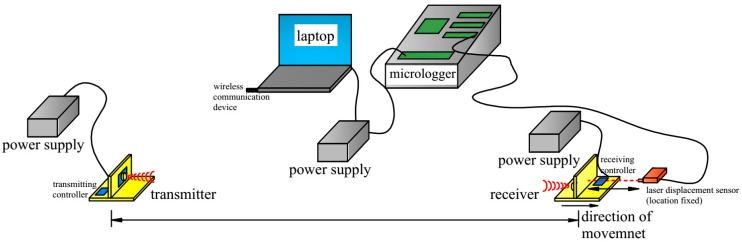
Experimental setup for evaluating the measurement accuracy of the ultrasonic transducers.

A calibration test was setup in order to examine the distance resolution measured by the ultrasonic transducers. The calibration setup consists of a pair of the ultrasonic transmitter and receiver, a Micro-Epsilon laser displacement sensor, a Campbell Scientific Micrologger, a laptop, and an electricity supply. The ILD 1302-100 laser displacement made by Micro-Epsilon has a measuring range between 50 mm and 150 mm, 20 μm resolution, and a source wavelength of 1550 nm. Its high resolution made it an ideal calibration device for the ultrasonic transducers. The laser sensor was connected to CR3000 Micrologger to record the data and then transmitted to the laptop. A wireless communication device was connected to the laptop, sending instructions from the laptop to the ultrasonic transducers. All the ultrasonic and laser sensors, the laptop, and the Micrologger were connected to power supplies (see Figure 6). The general idea of the experiment is to fix the sensors at a certain distance, angle, and misplacement; then, the distance was changed slightly (around 2 mm), several times. At each point, the distance was tested by ultrasonic three times, and the tiny change was calibrated by the high-accuracy Micro-Epsilon laser displacement sensor at a fixed location. The displacements measured by the ultrasonic were compared with those that were measured by the laser sensor.

### 3.2. Distance Measurement Accuracy Is Susceptible to Distance and Probe Angles

**Figure 7:**
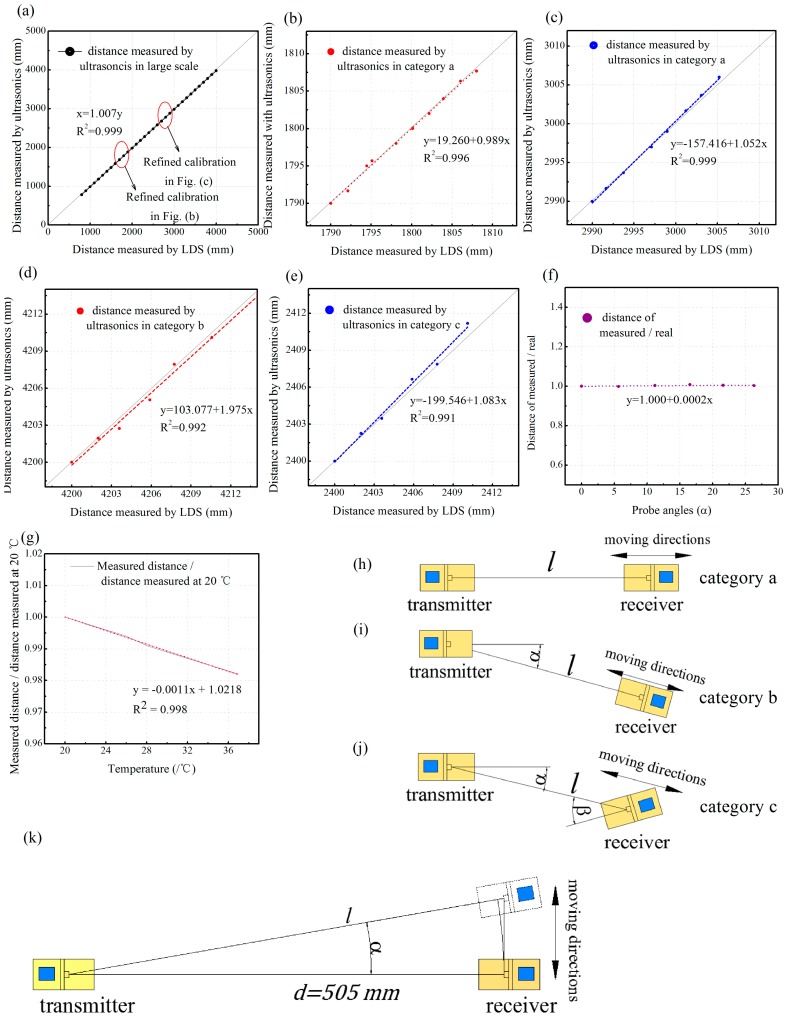
Accuracy of distance measurement with ultrasonic in large and small ranges under various distances, angles, and temperatures: (**a**) results of the large-range distance variations measured with a pair of ultrasonic transducers that were used against each other; (**b**) changes of distance measured with ultrasonic in category a (refer to Figure 1h) compared with those which were measured with the laser at around 1800 mm and (**c**) 3000 mm; (**d**) changes of distance measured with ultrasonic in category b of 4200 mm and (**e**) category c of 2400 mm; (**f**) the relationship between the normalized measured distance and the angle α (refer to Figure 1k); (**g**) the relationship between the normalized measured distance and the temperature (the ultrasonic sensors were dead against each other at a constant distance); (**h**–**j**) the relative position relationship of the sensor pair in category a, category b, and category c, respectively; (**k**) the schematic graph of tests setup in Figure 7f.

Several sets of tests were conducted to examine whether the ultrasonic system is qualified enough for measuring the micrometer-scale changes of the distance between the transmitter and receiver pairs, which may occur when the tunnel is deformed. To sum up, three key parameters that may result in a change of distance measured by the ultrasonic system are defined here, including the distance *l* between a transmitter-receiver pair (refer to Figure 7h), the angle *α* between the direction of the probe of the transmitter and the hypothetical line that connects the ultrasonic pair (refer to Figure 7i), and the angle *β* that the probe of the receiver deviates from the hypothetical connecting line of the ultrasonic pair (refer to Figure 7j). Accordingly, an ultrasonic transmitter-receiver pair was placed with varied *l*, *α*, *β* and then the distance *l* between the sensors was changed around 2 mm each time, several times, to verify whether the ultrasonic pair was capable enough to perceive micrometer-scale changes of distance under different relative location relationships. The distances were calibrated by a laser displacement sensor (LDS) with high accuracy. In addition, another set of test was performed under constant *l*, *β* (*β* = 0) but varied *α* to test the influence of angle *α* on the accuracy of the ultrasonic system.

In the first set of tests, an ultrasonic transmitter-receiver pair was placed on a line with angle *α* and *β* both equal to 0. The probes of the transmitter and the receiver were changed from 500 mm to 4000 mm. Results are shown in Figure 7a which indicates that the values measured by the ultrasonic transducers have a high linearity with the real distance (*R*^2^ = 0.999). This proves that the absolute distance measured by the ultrasonic transducer pair between around 0.5 m to 4.0 m is reliable and thus may suit for deformation monitoring of small to middle scale tunnel cross sections.

The micrometer-scale changes of distance between a pair of ultrasonic transducers that first fixed at some given distances were also tested to determine the distance measurement accuracy on a small scale. In total, five groups of tests were performed and the distances of the transmitter and the receiver probes around 1800 mm and 3000 mm were selected to conduct a more detailed calibration test. Results from Figure 7b,c indicated that the transducers could respond to changes in distance of less than 2 mm with a fair degree of accuracy. The analysis reveals that when a distance is within the range of at least 1 to 4 m, a change of distance of at most 2 mm can be perceived. Figure 7c shows an increase of deviation with the increase of distance; however, the deviation was no more than 0.8 mm at 3000 mm scale, and can be easily corrected by adding proper coefficients.

**Figure 8:**
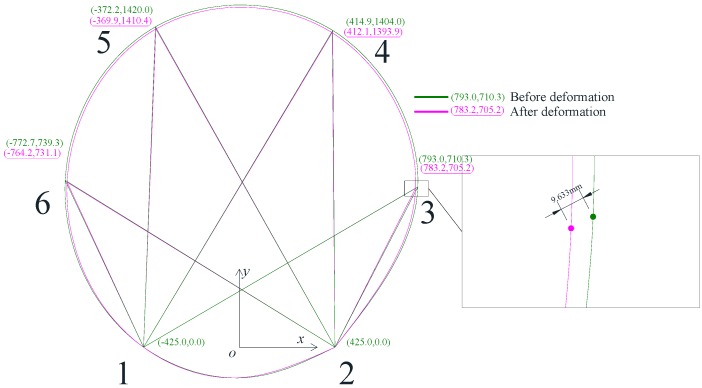
Deformation monitoring results of a 1.7 m-diameter tunnel model. Eight lengths between the ultrasonic transmitters and receivers were measured before and after deformation, and then the coordinates were calculated. Note that the points at the bottom of the tunnel were kept fixed throughout the test.

The performance of transducers may also be affected by the probe orientations or misplacements. As shown in Figure 7j, by considering a specific application in tunnel monitoring, there are two kinds of transmitter-receiver pair relative positions: (1) the probe of the receiver just oriented to the transmitter where the probe of transmitter has a 15° angle with the receiver (the sensor pair 1–3, 1–4, 2–5, and 2–6 in Figure 8 fall into this category); (2) the probe of the receiver has a 30° deviation to the transmitter and the probe of the transmitter has a 15° angle with the receiver (the sensor pair 1–5, 1–6, 2–3, and 2–4 in Figure 8 fall into this category). Thus, different configurations were set up to test whether the probe angles will lead to an inaccurate measurement.

Figure 7h–j shows the schematic graphs of all the three relative positions. It should be noted that during the tests, the angle *α* and *β* was kept constant, while only the *l* was changed. According to Figure 7d,e, when the transmitter and receiver were not just opposite each other but had an angle or were misplaced, the ultrasonic measured distances were still linear proportional to the real distances, and the deviations could be offset, according to the test data. The results show that even the transducer pair has an angle or misplacement between each other, and it could always perceive the change of distance as less as 2 mm. Another set of tests on the influence of varied angle *α* on the accuracy of distance measurement (refer to Figure 7k) was also tested, and the results are shown in Figure 7f. The distance of the transmitter-receiver pair was first set to *d* = 505 mm, then the receiver was placed on a line with varied angle *α* between the probes of the ultrasonic sensor pair. All the normalized measuring distances depicted on the graph are close to 1.0, which indicates that when a tunnel cross section confronts deformation that causes the change of transmitter-receiver probe-to-probe angle α, the distance measured with ultrasonic will still be reliable.

The temperature may also be a variable that influences the accuracy of ultrasonic distance measurement. The speed of ultrasonic goes up when the temperature increases, and this will result in the measured distance being less than the actual value. Thus, the temperature effect should be eliminated when there is a large temperature variation. An ultrasonic transmitter–receiver pair against each other at a constant distance was tested in a temperature varied from 20 to 37 °C. Figure 7g shows that when the temperature decreased, the measured distance decreased while the actual distances were constant, which was in accord with the nature property of ultrasonic. The data indicate an *R*^2^ = 0.998 linearity, and thus the temperature effect could be eliminated by adding the thermal transducer as well as setting the corresponding compensation formula. It should be noted that when applying the ultrasonic system for the physical model test in the lab, the temperature was kept constant, and it was hence unnecessary to take into account the influence of temperature in our experiments.

The above calibration test results indicate that this configuration of ultrasonic transducers can be applied in a small- to middle-size tunnel. The large deformations of a tunnel cross section (micrometer scale), such as large-scale convergence, can be calibrated by the ultrasonic system with a fair degree of accuracy.

### 3.3. Tunnel Deformation Measurement

The ultrasonic system was further verified in a physical tunnel model for deformation monitoring. The physical tunnel model was 1.7 m in diameter, which was fabricated in the laboratory. There were two ultrasonic transmitters and four ultrasonic receivers mounted on the tunnel model, as indicated in Figure 1. Points 1 and 2 at the bottom of the tunnel were fixed, while other parts of the tunnel were deformed such that the points at the sensors were displaced and then calibrated by LVDTs to 10 mm at each point. Figure 8 shows the results of the tunnel cross section before and after deformation. Eight lengths between ultrasonic transmitters and receivers were measured before and after deformation, and the coordinates at each ultrasonic sensor were calculated. Points and the coordinates before and after deformation were represented in the figure. As indicated in the figure, the displacements of sensors at points 3, 4, 5, and 6 were 11.810 mm, 9.872 mm, 10.481 mm, and 9.633 mm, respectively. By comparing with the LVDTs results, measurement errors at points 3, 4, 5, and 6 were 18.1%, 1.28%, 4.81%, and 3.67%, respectively. While there is a large measurement error for the sensor at point 3, the others were no more than 5% of the tunnel cross section deformation measurement. However, by considering larger tunnel dimensions in field situations, it is necessary to improve the measurement accuracy, which will be discussed in the following sections. It should be noticed that only the change of coordinates at each point, not the whole tunnel cross section, were monitored. Thus, a spinal curve was used to depict the whole tunnel cross section deformation, which may not firmly coincide with the real deformation.

**Figure 9:**
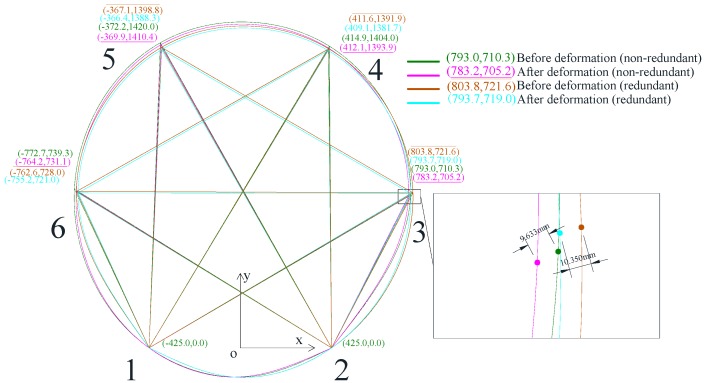
Results of the tunnel cross section deformation monitoring utilizing the RUI algorithm.

To apply the RUI method on the tunnel deformation monitoring system, two extra ultrasonic transmitters were used and mounted at point 7 and 8, as shown in Figure 2; thus, four more distances (*l*_74_, *l*_85_, *l*_78_, and *l*_87_) can be measured. With the RUI method, a total of 12 distances were measured and computed, as shown in Figure 9. The wave lagging errors was determined by considering the equations simultaneously, and the results are *e*_1_ = −10.0, *e*_2_ = −21.8, *e*_3_ = −13.1, *e*_4_ = 16.7 before deformation and *e*_1_ = −12.1, *e*_2_ = −21.0, *e*_3_ = −13.1, *e*_4_ = 14.9 after deformation. Without the redundant information, the changes of deformation at each point measured with ultrasonic were 11.8 mm, 9.9 mm, 10.5 mm, and 9.6 mm when the actual displacements were 10 mm. The average measurement error was 7%. By introducing RUI method, the changes of deformation at each point measured with ultrasonic were corrected to 10.2 mm, 10.5 mm, 10.5 mm, and 10.3 mm. The average measurement error was 3.75%. This analysis proves that by introducing RUI into the ultrasonic systems, a more accurate tunnel deformation monitoring was achieved and measurement errors were decreased.

## 4. Discussion

### 4.1. Influence of Distances, Probe Angles, and Temperatures on Measurement Accuracy

The measurement accuracy of the ultrasonic sensors with various distances was carefully examined, together with the influence of temperature and the change of the angles of transmitter–receiver.

(1) The test of the accuracy with varied measurement distances. One ultrasonic transmitter and one ultrasonic receiver were placed 800 mm opposite each other on a line, strictly. The transmitter was then controlled by the computer to emit three beams of ultrasonics with random intervals, and the durations between the beams emitted from the transmitter and the response from the receiver were recorded and converted into distances. Then, increasing the distance between the sensors by 100 mm per step, we recorded the distance measured by the ultrasonics transducers and LDS with maximum distance of 4000 mm. Results show that the ultrasonic transducers prove to have good linearity (*R*^2^ = 0.999) and accuracy of distance measurement.
(2) The resolutions of deformation measurement with varying distances, angles, and misplacements were further verified in the laboratory tests. For the deformation monitoring of a metro tunnel, the resolution of the ultrasonic system to the tiny changes of distance is more concerned than the absolute distance measured by the ultrasonic transducer. The results show that the ultrasonic transducers have the ability to capture distance variations of less than 2 mm.
(3) The changed probe angles during measurement was also investigated by considering two kinds of transmitter–receiver pair relative positions (as indicated in Figure 2 and Figure 4). One is the probe of the receiver, just oriented to the transmitter, but the probe of the transmitter has a 15° angle with the receiver. The other is when the probe of the receiver has a 30° deviation to the transmitter and the probe of the transmitter has a 15° angle with the receiver. To test whether these configurations will lead to an inaccurate measurement, a configuration that the transmitter was dead against the receiver was also tested. Calibration tests of the probe angles on ultrasonic distance measurements reveal that the ultrasonic measured distances were still linear proportional to the real ones, and the deviations can be offset by introducing proper coefficients. When the transducer pair has an angle or misplacement among itself, it could always perceive the change of distance as less than 2 mm.
(4) The influence of temperature on ultrasonic distance measurements was examined and the results indicate the distance measured by the ultrasonic transducers will be affected by the temperature. The measured values have a close linear relationship with temperature variations (*R*^2^ = 0.998). Thus, the temperature effect could be eliminated by adding the thermal transducer, as well as by setting the corresponding compensation formula.

### 4.2. Discussion of the Proposed RUI Wireless System

The main characteristic of the proposed wireless sensing system is its ability to provide a more accurate real-time tunnel deformation monitoring. The proposed approach overcomes the limitations of fixed measurement approaches and human measurements, providing an efficient tool to detect the tunnel deform, thus helping the safety control of in-service tunnels. This study highlights the role of the ultrasonic sensor arrangement in accurate measurement for a wireless tunnel deformation sensing system. We elucidated the effect of ultrasonic wave propagation angles, distances, and temperature changes on measurement accuracy, and derived an RUI approach for tunnel deformation measurements.

Previous studies have reported that the ultrasonic wave propagation distances and angles will diminish the measurement accuracy [15,16]. The present study indicates that the measurement accuracy can be achieved with different coefficients for various probe angles and sensor distances, as well as temperatures. The distance measured by the ultrasonic has a linear relationship with the measured results of laser displacement sensors, as shown in Figure 7. According to Figure 7a, with different distances, the measurement distances can be corrected by multiplying a linear coefficient of *c*_1_ = 1.007 to acquire the real distances. The temperature effects can also be eliminated from the measurements with the temperature compensation coefficient *C*_t_ = 1/(−0.0011t + 1.0218), where *t* is the temperature in Celsius. Another interesting finding is that the angle *α* does not appear to affect the measurement accuracy of the distances, as shown in Figure 7f. In other words, the measured distances are less affected by the probe angles *α*. Although there are limited test data, our findings indicate that the ultrasonic pairs can be used for wireless tunnel deformation measurement with high accuracy.

The lagging errors of ultrasonic wave propagation have been found to have highly affected the deformation measurement accuracy of the tunnel deformation, which was also reported by previous studies [17]. The present study puts forward the RUI approach in the tunnel deformation measurement using just two extra ultrasonic sensors. The average measurement error was decreased from 7% to 3.75%. Thus, the RUI approach can improve the measurement accuracy of the tunnel deformation by comparison with the approach without the RUI method.

However, due to the limited sensor points, only four point positions of a tunnel cross section can be measured; then, the whole tunnel cross section deformation was obtained with the interpolation, causing an inaccurate result between the measured points. While we normally do not need high accuracy of the distributed deformation of the tunnel cross section in the engineering practice, this could be resolved by using more ultrasonic received sensors, depending on the requirements of measurement accuracy. Despite the limited sensor points in a tunnel cross section, the ultrasonic sensor system with the RUI approach was successful in measuring the shapes of a tunnel cross section under arbitrary deformations.

## 5. Conclusions

In this study, we proposed a new progressing algorithm of the redundant ultrasonic information approach for an automatic and wireless tunnel deformation monitoring system with ultrasonic transducers. Details of the RUI approach were illustrated and applied to a physical model test. The experimental tests of the physical tunnel measured by the ultrasonic sensors with the RUI method were compared with the non-RUI approach. Results show that the average measurement error was decreased from 7% to 3.75% by including the RUI progressing algorithm.

The tunnel deformation will induce the variations of probe angles and distances of the ultrasonic sensors. Thus, a series of laboratory calibration tests were conducted on different probe angles, distances, and temperatures. Results show that measured results have a close linear relationship with the distance between the ultrasonic transmitter and the receiver sensors. The probe angles have less effect on the measured distances, while the various temperatures will affect the measured results. Therefore, the temperature effects should be eliminated from the measured results with the calibrated coefficient.

The proposed ultrasonic RUI monitoring system could provide a unique tool to study the metro tunnel deformations during constructions or operations. Compared with other approaches in metro tunnel deformation monitoring by human or total stations, the advantages of the proposed systems are: (1) wireless data transmission with real-time measurements; (2) obtaining a geometric shape of the tunnel deformation; (3) the easy implementation of the system in a tunnel cross section; and (4) the very low cost of the whole system. Therefore, the proposed system could be an effective tool for tunnel monitoring purposes.
